# Blood Biomarkers and Serologic Immunological Profiles Related to Periodontitis in Abdominal Aortic Aneurysm Patients

**DOI:** 10.3389/fcimb.2021.766462

**Published:** 2022-01-14

**Authors:** Leila Salhi, Patrick Rijkschroeff, Dorien Van Hede, Marja L. Laine, Wim Teughels, Natzi Sakalihasan, France Lambert

**Affiliations:** ^1^ Department of Periodontology, Buccal Surgery and Implantology, Faculty of Medicine, Liège, Belgium; ^2^ Department of Periodontology , Academic Centre for Dentistry Amsterdam, Vrije Universiteit (VU) Amsterdam, Amsterdam, Netherlands; ^3^ Department of Oral Health Sciences, KU Leuven & Dentistry, University Hospitals Leuven, Leuven, Belgium; ^4^ Department of Cardiovascular and Thoracic Surgery, Faculty of Medicine, Liège, Belgium

**Keywords:** periodontitis, periodontitis systemic interaction, microbiome, inflammation and innate immunity, abdominal aortic aneurysm (AAA)

## Abstract

**Background:**

Periodontitis is a chronic inflammatory gum disease associated with systemic diseases such as cardiovascular diseases.

**Aim:**

To investigate the association of systemic blood biomarkers, C-reactive protein (CRP), levels of lipopolysaccharide (LPS), and IgG levels against periodontal pathogens *Aggregatibacter actinomycetemcomitans* (Aa) and *Porphyromonas gingivalis* (Pg) with the stability, based on the aortic diameter, the growth rate and the eligibility for surgical intervention, of patients with abdominal aortic aneurysm (AAA).

**Methods:**

Patients with stable AAA (n = 30) and unstable AAA (n = 31) were recruited. The anti-*A. actinomycetemcomitans* and anti-*P. gingivalis* IgG levels were analyzed by ELISA, the LPS analysis was performed by using the limulus amebocyte lysate (LAL) test, and plasma levels of CRP were determined using an immune turbidimetric method. The association between these blood systemic biomarkers, AAA features, periodontal clinical parameters and oral microbial profiles were explored. Regression models were used to test the relationship between variables.

**Results:**

The presence of antibodies against Pg and Aa, LPS and high CRP concentrations were found in all AAA patients. The IgG levels were similar in patients with stable and unstable AAA (both for Aa and Pg). Among investigated blood biomarkers, only CRP was associated with AAA stability. The amount of LPS in saliva, supra, and subgingival plaque were significantly associated with the systemic LPS (p <0.05).

**Conclusions:**

This *post-hoc* study emphasizes the presence of antibodies against Pg and Aa, LPS and high CRP concentrations in all AAA patients. The presence of Pg in saliva and subgingival plaque was significantly associated with the blood LPS levels. For further studies investigating periodontitis and systemic diseases, specific predictive blood biomarkers should be considered instead of the use of antibodies alone.

## Introduction

AAA is a chronic degenerative disorder of the abdominal aorta promoted by genetic and environmental risk factors such as smoking, older age, Caucasian ethnicity, and the male gender (Sakalihasan et al., 2018). The disease progresses with the increase of the abdominal aortic diameter that can lead to vessel rupture, responsible for 1 to 3% of all deaths in western countries (Sakalihasan et al., 2005). The AAA physiopathology involves an inflammatory destruction of the aortic wall structure and the presence of an intraluminal thrombus (ILT) (Hellmann et al., 2007; Morbelli et al., 2014) that can be induced by the presence of several bacteria (Halme et al., 1999; Lindholt et al., 1999; Salhi et al., 2019).

Periodontitis, a chronic inflammatory gum pathology, is the 6th most prevalent disease worldwide (Kassebaum et al., 2014) affecting more than 50% of the adult population and about 11% of the population suffers from severe form. The disease, induced by the invasion of gram negative bacteria (Socransky et al., 1998), triggering the host immune defense (Haffajee and Socransky, 1994; Amano, 2010a; Meyle and Chapple, 2015), leads to the destruction of the connective tissues supporting tooth, and ultimately to the tooth loss (Socransky et al., 1998; Meyle and Chapple, 2015). Particularly, the red complex composed of *Treponema denticola (Td)*, *Porphyromonas gingivalis* (*Pg)*, *Tannarella forsythia (Tf)*, and *Aggregatibacter actinomycetemcomitans (Aa)* have been strongly associated with periodontal tissues destruction (Haffajee and Socransky, 1994; Amano, 2010a; Meyle and Chapple, 2015). Moreover, it is well documented that the release of periodontal pathogens or/and their sub-products (SPs) (Forner et al., 2006) in the bloodstream (Haffajee and Socransky, 1994) can induce a systemic inflammation and promote metastatic infection of periodontal pathogens (Loos, 2005; Salhi et al., 2019). Therefore, it has been hypothesized that periodontitis may induce bacteremia, the release of inflammatory mediators and the progression of systemic diseases (Forner et al., 2006; Amano, 2010a). Among these inflammatory mediators released by Pg, hemagglutinin can promote the platelet aggregation (Belanger et al., 2012) and gingipains can neutralized the host immune response extra-orally, in a metastatic site (Nassar et al., 2002). Other virulence factors such as the lipopolysaccharide (Bainbridge et al., 2002) (LPS) of *Pg* or the cytotoxin of *Aa* (Tan et al., 2002), can also impair systemic diseases, as CVD by disturbing the immune host response (Deshpande et al., 1998a; Deshpande et al., 1998b; Amano, 2010a). The microbial properties of specific periodontal pathogens contribute to the development of chronic non-communicable disease such as cardiovascular diseases (CVD) (Loos et al., 2000; Tonetti and Van Dyke, 2013) and diabetes (Tonetti and Van Dyke, 2013; Sanz et al., 2018). Indeed, serum antibodies against periodontal pathogens and LPS were shown to be associated with higher risk for ischemic stroke (Pussinen et al., 2004a; Tabeta et al., 2011; Hosomi et al., 2012; Palm et al., 2014), coronary heart disease (Pussinen et al., 2003; Pussinen et al., 2005; Goteiner et al., 2008) as myocardial infarction (Pussinen et al., 2004b). Therefore, in patients suffering from CVD as AAA, the concentrations of immunoglobulin G (IgG) against periodontal bacteria (Nakagawa et al., 1994; Albandar et al., 2001; Dye et al., 2009; Pussinen et al., 2011), LPS (Ebersole et al., 2010) and C-reactive protein (CRP) might be relevant periodontitis-related blood biomarkers to characterize sequelae linked to periodontitis.

The aim of this study was to investigate the relationship between blood biomarkers and serologic immunological profiles related to periodontitis (CRP, LPS, and serum anti- *Aggregatibacter actinomycetemcomitans* [*Aa*] and anti-*Porphyromonas gingivalis* [*Pg*] IgG) in abdominal aortic aneurysm patients. Secondary objectives focused on the relationship between periodontal clinical parameters, microbial profile and periodontitis-related blood biomarkers.

## Material and Methods

### Study Design and Ethical Committee

Unexploited data collected during a previous cross-sectional study on the periodontitis and AAA (Salhi et al., 2020) were used in the present *post-hoc* study to further explore the associations between AAA stability and periodontitis related blood biomarkers. The study design, sample size calculation, patient selection, and demographics of the cohort have been described in the previous report (Salhi et al., 2020). AAA imaging, periodontal, and microbiological parameters are briefly explained hereafter.

The study was approved by the human subjects’ ethics board of the University Hospital of Liege, Belgium (B707201421977), and was registered on clinicaltrial.gov (file number: NCT03767023).

### Clinical Data and Blood Sample Collection

After screening the medical files of eligible AAA patients, the participants were invited to the Department of Periodontology where anamnesis and imaging were recorded. A full periodontal clinical and microbiological examination was performed by a single investigator (LS) and the blood samples were collected by the department nurse (JN M). The included AAA patients were divided into two groups according to the stability of the AAA, defined by Sweeting et al. (2012). The first group consisted of patients with stable AAA (n = 30), characterized by an antero-posterior abdominal diameter inferior to 55 mm and/or a stable growth rate inferior to 10 mm per year and not considered for surgical repair. The second group was composed of patients with an unstable AAA (n = 31) with a diameter higher than 55 mm and rapid growth rate superior to 10 mm per year), requiring short-term open surgery or endovascular aneurismal repair. The AAA growth rate was estimated from the previous AAA diameter recordings found in the patient file (at least 2 exams at a given time).

### AAA Imaging Data

The AAA dimensions were assessed by measuring AAA diameters (anterior–posterior, cross-sectional, and maximal) and volumes (entire AAA, residual lumen, and thrombus), as described previously (Salhi et al., 2020). More precisely, the AAA diameters (mm) were collected in the medical file of the patient according to the last diameter based on echography (images obtained with a C5.2 convex probe, IU22 Philips Ultrasonography, Belgium) for 9 patients or computed tomography (CT) scan for 52 patients. When abdominal CT scans (slides thickness: 1.25 mm) were available, additional measurements such as the AAA diameters and volumes were collected by two independent blinded and calibrated examiners (LS and AG) using a specific imaging software (Syngovia by Siemens Healthineers, Erlangen, Germany). Aortic diameters (mm) were measured in their anterior–posterior, cross-sectional, and maximal positions based on axial acquisitions. The volumes of the entire AAA (mm³) and the thrombus were measured by using the VOI free hands tool. The lumen volume was obtained by the subtraction of the entire AAA volume and the residual lumen volume. AAA heights (mm) were recorded from the iliac bifurcation to the renal artery origin and to the neck of the AAA, respectively.

### Periodontal Data

The number of teeth, the presence of healthy gingiva or gingivitis, diagnosis, and classification of periodontitis (stage, extent, and grade) were recorded for each subject according to Caton et al. (2018). Periodontal parameters were collected by a single periodontist (LS) including pocket probing depth (PD, mm; 6 sites per tooth), gingival recession (RD, mm), clinical attachment level (CAL, mm), bleeding on probing (Silness and Loe, 1964) (BOP, %), percentage (%) of PD sites ≥6 mm calculated over all PD sites of the patient, plaque score index (PI, %), furcations (Hamp et al., 1975) and tooth mobility (Miller, 1938). A graduated manual periodontal probe^1^ was used to measure (mm) 6 sites per tooth. The Periodontal Inflamed Surface Area (PISA), the global PISA score (Nesse et al., 2008) and the Periodontal Index for Risk of Infectiousness (PIRI) (Rompen et al., 2001), which takes into account the number and the severity of the periodontal niches in contact with blood circulation (PD and furcation impairment), were calculated. Scores were attributed to the number and depth of PD and for the number and severity of furcations (Rompen et al., 2001). By adding these 2 scores, patients were classified according their risk for metastatic injury: none or low risk (PIRI = 0), moderate risk (1 ≤PIRI≤ 5), and high risk (6 ≤PIRI≤ 10).

### Microbiological Data

All microbiological samples were collected by the same investigator (LS). Saliva samples were obtained by collecting 500 µl to 1 ml of unstimulated saliva. The supragingival samples were collected from the four teeth with the deepest periodontal pockets. The sites were isolated with cotton rolls and gently dried with compressed air (Teughels et al., 2013). All supragingival plaque was taken with a periodontal curette and then placed in a sterile tube containing 0.75 ml of TE (10 mM Tris–HCl, 1 mM EDTA, pH 7.6) and an equal amount of 0.5 M NaOH. Then, the subgingival samples were harvested at the same four deepest pocket sites. The harvesting was performed by using a sterile endo paperpoint inserted into the pocket (Iso 040, Dentsply, Maillefer, Switzerland). Four paperpoints were inserted per pocket for 15 s (16 tips per patient), and were then collected as for supragingival samples. All samples were stored at −20°C. After defrosting and vigorously vortexing, 400 µl of each sample were centrifuged at 13,000*g*. The obtained pellets were dispersed in 200 µl Instagen. DNA was extracted with InstaGene matrix (Bio-Rad Life Science Research, Hercules, CA, USA) according to the instructions of the manufacturer. Five microliters of the purified DNA were used for the detection and quantification of *Tannerella forsythia* (Tf), *Porphyromonas gingivalis* (Pg), *Aggregatibacter actinomycetemcomitans* (Aa), *Fusobacterium nucleatum* (Fn), and *Prevotella intermedia* (Pi) by real time quantitative polymerase chain reaction (RT-qPCR). The RT-qPCR assay was performed with a CFX96 Real-Time System^2^ using a Taqman 5’ nuclease assay PCR method for detection and quantification of bacterial DNA. Assay conditions for all primer/probe sets consisted of an initial 2 min at 50°C, followed by a denaturation step for 10 min at 95°C, followed by 45 cycles of 95°C for 15 s and 60°C for 60 s. Quantification was based on a plasmid standard curve (PMID: 19930094). Results were expressed as log 10 genome equivalents (gEq)/ml. Values below the detection threshold level were recorded as 0.

### Periodontitis-Related Blood Biomarkers Data

In order to induce bacteremia in blood circulation, patients were invited to have a standardized mastication on paraffin (Nicu et al., 2009) and to brush their teeth during 2 min. Afterwards, venous blood samples from antecubital fossa were taken to evaluate the blood biomarkers of interest.

### IgG Against Aa and Pg

A first blood sample (10 ml red cap) was harvested to quantify the concentration of antibodies levels against *Pg* and *Aa*. The tube was kept at room temperature for 30 min and then the serum was obtained by centrifugation at 2,000*g* at 4°C for 10 min. Aliquots of serum were stored at −80°C. The antibodies against *Pg* and *Aa* were analyzed as previously described (Nicu et al., 2009). Briefly, a mixture was made out of five different strains of *Aa* (ATCC 29523, Y4, NCTC9710, 3381, and OM2 534 representing the serotypes a, b, c, d, and e) for the detection of IgG levels against *Aa*; and 8 different strains of *Pg* (W83, HG 184, A7A1-28, ATCC 49417, HG 1690, HG 1691 and 34-4 representing the capsule serotypes K1–K7, and also the uncapsulated strain 381 (Laine et al., 1997) for the detection of IgG levels against *Pg*. The *Aa* strains were grown for 18 h in brain–heart-infusion (BHI) broth (Sigma Chemical Co, St. Louis, MO, USA) aerobically at 37°C in humidified 5% CO_2_. The *Pg* strains were grown anaerobically (80% N_2_, 10% H_2_, 10% CO_2_) at 37°C for 18 h in BHI broth supplemented with hemin (5 mg/l) and menadione (1 mg/l) (Sigma). The bacteria were washed once with phosphate-buffered saline (PBS; 10 mM phosphate, 150 mM NaCl, pH 7.4) and then fixed overnight at 4°C in 0.5% paraformaldehyde–PBS. Further, the bacterial suspensions were washed three times in PBS, sonicated and brought to an optical density corresponding to an absorbance of 0.15 at 580 nm in ELISA-buffer (PBS, 0.5% bovine serum albumin, 0.05% Tween-20). For ELISA, 150 ml of the sonicated mixture from the five *Aa* or from the eight *Pg* strains were used to coat Microlon ELISA plates (Greiner Bio-One B.V., Alphen a/d Rijn, the Netherlands). The unspecific bindings were blocked by 5% bovine serum albumin (BSA) in PBS at room temperature for 30 min. Diluted (1:1,500) serum samples were tested in duplicate. The plates were incubated for 2 h at room temperature and washed three times in ELISA buffer. Horse-radish peroxidase-conjugated, goat anti-human IgG (Vector Laboratories Inc., Burlingame, CA, USA) diluted (1:2,000; 150 ml) were then added and the plates were incubated for 2 h at room temperature. Substrate was then added and absorbance values were measured at 450 nm with a multilabel counter (Wallac Victor 1420, Perkin-Elmer Life Sciences, Boston, MA, USA).

### Endotoxin Detection Assay

A second tube of blood (10 ml EDTA plasma tube) was collected for LPS analysis and immediately placed on ice. Plasma was obtained by centrifugation at 2,000*g* at 4°C for 10 min. Aliquots of plasma were stored at −80°C. The LPS analysis was performed by using the EndoLISA kit according to the manufacturer’s instructions (EndoLISA^®^, Hyglos GmbH, Biomérieux, Bernried am Starnberger See, Germany). Briefly, all samples were defrosted and vortexed to ensure homogeneity. CSE (endotoxin standard *Escherichia coli* O55:B5) was diluted for the standard curve with dilution factor 10× and dissolved in endotoxin-free water. In total, 100 µl of each preparation was added in duplicate into the respective wells. A blank control was included as a negative control. Next, 20 µl of 6× Binding buffer was added to each well and was covered in aluminum foil and incubated at 37°C for 90 min at 450 rpm. Samples were washed 3× with 150 µl Wash Buffer before 100 µl of the EndoLISA assay reagent was added to each well. Fluorescent signals were measured at 37°C immediately and after 90 min.

### CRP

A third blood sample (lithium-heparinate) was used for plasma cell analysis and for high sensitivity CRP (hsCRP), using a commercially available kit (Behring N latex C-reactive protein mono Analyzer, Behring Diagnostic, Marburg, Germany). This sample was analyzed within 3 h in a clinical chemistry laboratory, by using standardized and automated procedures.

### Statistical Methods

The power calculation of the larger study of which this one is a *post-hoc* analysis showed that at least 30 patients had to be included in both stable and unstable AAA groups. Results were expressed as mean ± SD for quantitative variables and as frequency tables (numbers and percentage) for categorical variables. Non-normal distributed variables were log-transformed to normalize their distribution. Comparisons between stable and unstable AAA groups were done by Student’s *t*-test or Kruskal–Wallis test for continuous variables and by chi-square test or Fisher exact test for qualitative findings. Regression models were used to test the relationship between study variables and results were expressed as regression coefficients and their standard error (SE). A positive or negative regression coefficient indicated respectively an increasing and a decreasing relationship between the two variables. All regressions were adjusted for AAA stability. When the dependent variable Y was quantitative, classical linear regression was applied. When Y was binary, the logistic regression was used and when Y was ordinal then ordinal logistic regression was applied. Results were considered significant at the 5% level (*p <*0.05). Calculations were done in SAS (SAS Institute, Cary, NC) version 9.4.

## Results

The periodontal characteristics of study patients with stable (n = 30) or unstable (n = 31) AAA were previously described (Salhi et al., 2020).

The correlation (in terms of regression coefficients) between clinical periodontal parameters, CRP and LPS blood levels, and antibodies concentrations (anti-Aa and anti-Pg) are presented in **Table 1** No correlation was found neither with CRP nor with LPS whereas a trend was found between BOP and anti-Aa antibodies (*p* = 0.05).

**Table 1:** Relationship between periodontal parameters and the blood concentrations of CRP and LPS, *Aa*, and *Pg* antibodies.

	C-reactive protein^(a)^	Lipopolysaccharide^(a)^	anti-*Aggregatibacter actinomycetemcomitans* ^(a)^	anti-*Porphyromonas gingivalis* ^(a)^
	Coefficient ± SE^(e)^	Coefficient ± SE^(e)^	Coefficient ± SE^(e)^	Coefficient ± SE^(e)^
PISA	0.64 ± 1.20	0.17 ± 0.23	0.17 ± 0.16	0.038 ± 0.21
PD mean	−0.058 ± 0.088	−0.027 ± 0.017	0.013 ± 0.011	−0.0078 ± 0.015
PD max	0.026 ± 0.18	−0.040 ± 0.036	−0.022 ± 0.025	−0.021 ± 0.033
BOP	−1.00 ± 3.66	0.89 ± 0.71	1.00 ± 0.47**	0.092 ± 0.65
%PD > 6 mm	−0.21 ± 0.21	−0.034 ± 0.040	0.016 ± 0.028	−0.024 ± 0.036
Periodontitis^(c)^				
Stage	0.032 ± 0.21	−0.080 ± 0.044*	−0.0007 ± 0.028	0.028 ± 0.037
Extent M-I vs L	0.60 ± 0.51	0.19 ± 0.16	0.0031 ± 0.080	−0.16 ± 0.12
Extent G vs L	0.0002 ± 0.28	−0.086 ± 0.071	0.049 ± 0.038	−0.019 ± 0.048
Grade	−0.20 ± 0.23	−0.074 ± 0.047	0.0050 ± 0.030	−0.016 ± 0.039
PIRI score	−0.012 ± 0.29	−0.10 ± 0.057*	−0.045 ± 0.040	0.021 ± 0.053
PIRI category	0.15 ± 0.22	−0.031 ± 0.043	−0.020 ± 0.030	0.064 ± 0.041
Furcation site^(d)^	−0.066 ± 0.23	−0.11 ± 0.059*	−0.059 ± 0.036	0.035 ± 0.043
Mobility site^(d)^	−0.23 ± 0.22	−0.035 ± 0.041	−0.0013 ± 0.028	0.016 ± 0.037

Aa, Aggregatibacter actinomycetemcomitans; Pg, Porphyromonas gingivalis; CRP, C-reactive protein; LPS, lipopolysaccharide; PISA, periodontal inflamed surface area; PD, pocket depth; BOP, bleeding on probing; PIRI, periodontal index for risk of infectiousness.

*Tendency (p < 0.10); **significant (p < 0.05).

(a) Log-transform applied to antibodies concentration, CRP and LPS.

PISA, periodontal inflamed surface area; PD, pocket depth; BOP, bleeding on probing; PIRI, periodontal index for risk of infectiousness.

(c) Ordinal logistic regression for stage and grade; multinomial logistic for extent.

(d) Ordinal logistic regression.

(e) All regression coefficients were adjusted for group (stable/unstable); a positive (negative) coefficient indicates an increasing (decreasing) relationship between antibodies, CRP or LPS and periodontal clinical parameters.

The periodontitis-related blood biomarkers analysis according to AAA stability are shown in **Table 2**. The CRP levels were higher in patients with unstable AAA (*p* = 0.017) while LPS levels did not differ between the groups. Anti-Aa and anti-Pg antibodies were found in similar concentrations in all blood samples of patients with stable and unstable AAA. The relationship between periodontal-specific blood markers, AAA diameters and volumes are shown in **Table 3**. No significant associations were found.

**Table 2:** Blood markers analysis of study patients with stable or unstable AAA.

Variable	Stable AAA	Unstable AAA	P-value
	*N* = 30	*N* = 31	
CRP (mg/ml)	5.71 ± 18.40	5.83 ± 6.20	0.02
LPS (pg/ml)	334 ± 1736	46.60 ± 111	0.44
Antibodies (OD_450 nm_)*			
Anti-Aa	18.50 ± 9.89	14.90 ± 8.10	0.14
Anti-Pg	12.30 ± 7.00	9.07 ± 7.05	0.08

CRP, C-reactive protein; LPS, lipopolysaccharide; Aa, Aggregatibacter actinomycetemcomitans; Pg, Porphyromonas gingivalis.

**Table 3:** Relationship between the blood concentrations of CRP, LPS, *Aa*, and *Pg* antibodies, and the AAA diameters and volumes (*N* = 57).

	AAA diameter ^(d)^			AAA volume ^(d)^		
	Antero-posterior	Cross-sectional	Maximal	Aneurysm	Residual lumen	Thrombus
	Coefficient ± SE ^(e)^			Coefficient ± SE ^(e)^		
CRP ^(a)^	0.41 ± 0.85	0.006 ± 0.82	0.78 ± 0.99	0.19 ± 0.30	−0.20 ± 0.30	0.27 ± 0.26
LPS ^(b)^	0.16 ± 5.70	2.01 ± 5.22	−2.17 ± 5.88	1.12 ± 1.96	1.05 ± 1.45	−1.33 ± 1.70
Anti-*Aa* ^(c)^	11.5 ± 7.99	9.81 ± 7.33	11.2 ± 8.46	4.27 ± 2.73	3.19 ± 2.62	1.10 ± 2.39
Anti-*Pg* ^(c)^	-3.52 ± 6.47	-5.39 ± 5.90	-4.28 ± 6.50	−2.04 ± 2.21	−3.30 ± 2.07	−1.60 ± 1.83

Aa, Aggregatibacter actinomycetemcomitans; Pg, Porphyromonas gingivalis; CRP, C-reactive protein; LPS, lipopolysaccharide; AAA, abdominal aortic aneurysm.

(a) CRP level expressed in log10 of no. of proteins (mg/ml)

(b) LPS amount expressed in log10 of no. of endotoxin (LPS/ml).

(c) All antibodies amounts expressed in log10 of no. of genome equivalents (Geq/ml).

(d) AAA diameters and volumes log-transformed.

(e) Regression coefficients derived by linear regression adjusted for group (stable/unstable); a positive (negative) coefficient indicates an increasing (decreasing) relationship between pathogens and diameters/volumes.

The relationships between Aa and Pg in saliva, supra-gingival plaque and subgingival plaque and the levels of CRP, LPS and anti-Aa/anti-Pg antibodies in blood are shown in **Table 4**. The presence of Pg in saliva and subgingival plaque was correlated to the LPS blood levels (p <0.05). No other significant associations were found between microbial profiles and blood biomarkers.

**Table 4:** Relationship between periodontal pathogens in saliva, supragingival plaque, and subgingival plaque with the concentrations of the Aa and Pg antibodies, CRP and LPS.

		[CRP]^(b)^	[LPS]^(b)^	[anti-*Aa*] ^(b)^	[anti-*Pg*]^(b)^
Sample ^(a)^	Pathogen	Coefficient ± SE ^(c)^	Coefficient ± SE ^(c)^	Coefficient ± SE ^(c)^	Coefficient ± SE ^(c)^
Saliva	*Aa*	−0.034 ± 0.13	−0.007 ± 0.028	−0.0095 ± 0.017	–
	*Pg*	−0.075 ± 0.15	0.076 ± 0.035*	–	0.017 ± 0.026
Supragingival	*Aa*	0.066 ± 0.10	−0.029 ± 0.027	−0.017 ± 0.015	–
	*Pg*	−0.084 ± 0.15	0.062 ± 0.033	–	0.030 ± 0.027
Subgingival	*Aa*	−0.17 ± 0.12	−0.035 ± 0.029	−0.0049 ± 0.017	–
	*Pg*	−0.18 ± 0.15	0.071 ± 0.034*	–	0.014 ± 0.026

CRP, C-reactive protein; LPS, lipopolysaccharide; Aa, Aggregatibacter actinomycetemcomitans; Pg, Porphyromonas gingivalis.

Significant *(p < 0.05).

(a) All periodonpathogens amounts expressed in log10 of number of genome equivalents (Geq/ml).

(b) Log-transform applied to antibodies concentration, CRP and LPS.

(c) Regression coefficients derived by linear regression adjusted for group (stable/unstable); a positive (negative) coefficient indicates an increasing (decreasing) relationship between pathogens and CRP, LPS and antibodies amount.

## Discussion

This *post-hoc* study of a cross-sectional, non-interventional, study focused on the relationship between periodontitis-specific blood biomarkers and the AAA stability.

Strikingly, all the included patients presented periodontitis and periodontitis-specific blood markers (antibodies against the periodontal pathogens *Pg* and *Aa* and also LPS and CRP) although periodontitis was not an inclusion criterion of the study. Additionally, the LPS in blood was associated with the presence of Pg in saliva and subgingival plaque. Therefore, it seems that periodontitis may have induced bacteremia, endotoxemia and systemic inflammation which may eventually have a role in the physiopathology of AAA (Mealey et al., 1999).

In a previous publication, the association between the severity of the periodontal parameters, the quantity of periodontal pathogens and the severity (or the extend) of AAA was observed (Salhi et al., 2020) and the present serologic immunological profiles support these findings. Although IgG antibodies against *Pg* and *Aa* were detected in all patients, no association was found with the concentration of bacteria in subgingival plaque samples. The low levels of IgG measured in the serum may reflect an altered immune response that may contribute to the physiopathology of periodontitis and AAA (Kuivaniemi et al., 2015; Sakalihasan et al., 2018). Therefore, the quantification of seral anti-bacterial antibodies remains controversial in the diagnosis of past or current periodontitis exposure (Papapanou et al., 2000; Dye et al., 2009; Vlachojannis et al., 2010; Pussinen et al., 2011), namely due to the possible cross-reactions with other bacterial epitopes (Davison et al., 2021). Thus, the antibody titers, at least as a single marker, may not be sufficient to explore the potential association between periodontitis and CVD.

Hence, in addition to IgG detection in sera, further seral investigations should be considered, as the use of bacteria virulence (Amano, 2010b) factors. Indeed, as suggested by some authors (Loos et al., 2000; Tonetti and Van Dyke, 2013), the detection and quantification of additional markers such as fimbriae, gingipains and hemagglutinin (Amano, 2010a) and also bacteria toxins would be relevant to further understand the systemic effect of periodontitis on chronic non-communicable diseases as cardiovascular diseases (CVD) including AAA. In the present study, antibodies against *Pg* tended to be slightly lower in unstable AAA patients when compared to those of patients in stable AAA (p = 0.08). It could be hypothesized that virulence factors of *Pg* such as gingipains (Haruyama et al., 2009), fimbriae (Fan et al., 2001), capsule (Laine et al., 1997), and LPS (Bainbridge and Darveau, 2001) contribute to escape from the host immune response, lowering IgG detection in serum and, finally, contributing to periodontitis progression (Hajishengallis et al., 2012). A second interpretation could be found in the high number of tooth losses of the included patients suffering from severe form of periodontitis. Indeed, as recently suggested, the lower antibody titers against *Pg* were associated with an increased number of tooth loss (Aoyama et al., 2018). Therefore, tooth loss may reflect the end stage of periodontitis (Caton et al., 2018) and has been often associated with cardiovascular events (Liljestrand et al., 2015) such as myocardial infarction and stroke (Lee et al., 2019).

Additionally, the study also showed that patients with unstable AAA displayed higher CRP levels, a blood biomarker usually associated with the progression of cardiovascular disease (Loos, 2005). Although CRP is a non-specific biomarker, the present findings suggest that periodontitis may contribute to the elevated CRP concentrations and support the relationship between periodontitis, inflammation, and AAA instability. Indeed, the augmentation of systemic markers due to periodontitis (D’Aiuto et al., 2007; Paraskevas et al., 2008; Lima et al., 2011; Fedele et al., 2011; Nibali et al., 2013; Shaddox et al., 2013; Balli et al., 2014; Keles et al., 2014; Finoti et al., 2017; Chandy et al., 2017; Batschkus et al., 2017), such as specific interleukins, fibrinogen, albumin, CRP, matrix metalloproteinase-9 or tumor necrosis factor-α, participate to the vascular endothelial weakening and its dysfunction (Tonetti et al., 2007) and, therefore, can promote systemic diseases. Thus, the presence of periodontitis may enhance systemic inflammation which is involved in the AAA physiopathology (Tambyraja et al., 2007; Wallinder et al., 2009; Courtois et al., 2013; Martinez-Pinna et al., 2013; Morbelli et al., 2014).

This study suffers from some limitations, particularly because of the *post-hoc* design and the small sample size. Including a control group with healthy patient (without AAA), would also be of interest in future research. Findings should therefore be interpreted cautiously.

### Conclusion

This *post-hoc* study emphasizes the presence of antibodies against Pg and Aa, LPS and high CRP concentrations in all AAA patients. However, among investigated blood biomarkers, only CRP was associated with AAA stability. The presence of Pg in saliva and subgingival plaque was significantly associated with the blood LPS levels. For further studies investigating periodontitis and systemic diseases, specific predictive blood biomarkers should be considered instead of the use of antibodies alone.

## Data Availability Statement

The raw data supporting the conclusions of this article will be made available by the authors, without undue reservation.

## Ethics Statement

The studies involving human participants were reviewed and approved by the human subjects’ ethics board of the University Hospital of Liege, Belgium (B707201421977). The patients/participants provided their written informed consent to participate in this study. Written informed consent was obtained from the individual(s) for the publication of any potentially identifiable images or data included in this article.

## Author Contributions

Conceptualization (LS, FL); Investigation (LS); Methodology (LS, PR, WT); Supervision (ML, FL); Roles/Writing - original draft (LS); Writing – review (LS, PR, ML, DV, FL) Validation (LS, PR, DV, ML, WT, NS, FL).

## Funding

The authors received no financial support for the research, authorship, and/or publication of this article.

## Conflict of Interest

The authors declare that the research was conducted in the absence of any commercial or financial relationships that could be construed as a potential conflict of interest.

## Publisher’s Note

All claims expressed in this article are solely those of the authors and do not necessarily represent those of their affiliated organizations, or those of the publisher, the editors and the reviewers. Any product that may be evaluated in this article, or claim that may be made by its manufacturer, is not guaranteed or endorsed by the publisher.

## Footnotes

1 Periodontal probe, North Carolina 2927.10, Stoma, Germany

2 BioRad, Hercules, California, United-States
